# Genetic analysis and management of a familial hypercholesterolemia pedigree with polygenic variants: Case report

**DOI:** 10.1097/MD.0000000000034534

**Published:** 2023-08-11

**Authors:** Yu Han, Lin Zhang, Huimin Tao, Jiebin Wu, Jingfang Zhai

**Affiliations:** a Affiliated Xuzhou Clinical College of Xuzhou Medical University, Xuzhou, China; b Key Laboratory of Brain Diseases Bioinformation of Xuzhou Medical University, Xuzhou, China; c Department of Prenatal Diagnosis Medical Center, Xuzhou Central Hospital, Xuzhou, China; d School of Information and Control Engineering, China University of Mining and Technology, Xuzhou, China.

**Keywords:** familial hypercholesterolemia, LDLR gene, pedigree whole exome sequencing

## Abstract

Familial hypercholesterolemia (FH) is an autosomal dominant genetic disorder typically caused by low density lipoprotein receptor (LDLR) gene mutation. Herein, we reported a FH pedigree with polygenic variants: LDLR, apolipoprotein B (APOB), and epoxide hydrolase 2 (EPHX2).

**Patient concerns:**

A 10-year-old boy mainly presented multiple skin xanthomas and hypercholesterolemia. His family visited our hospital and was performed with pedigree whole exome sequencing (WES) at 20 + 3 weeks gestation of the mother’s second pregnancy.

**Diagnoses:**

Based on the clinical features and genetic analysis, the pedigree was diagnosed with familial hypercholesterolemia.

**Interventions:**

After genetic counseling, the couple opted to continue the pregnancy. Treatment advice and follow-up were offered to them.

**Outcomes:**

A novel compound heterozygous LDLR mutation: c.1009G>T and c.68-2A>G, derived from his parents respectively was revealed through pedigree WES, meanwhile, a maternal APOB gene variant: c.1670A>G and a paternal EPHX2 gene variant: c.548 dup of the proband were found together. Furthermore, the same compound heterozygous LDLR mutation as his was confirmed in his sister without APOB and EPHX2 variants in her fetal stage.

**Lessons:**

WES combined with clinical features is essential for the diagnosis of FH, however, prenatal genetic testing results might bring more challenges to prenatal genetic counseling. Furthermore, it is more important to provide the guidance and early intervention for such families in the long run.

## 1. Introduction

Familial hypercholesterolemia (FH, OMIM 143890) is an autosomal dominant genetic disorder characterized by skin xanthomas and atherosclerotic cardiovascular disease (ASCVD) due to the increase of low density lipoprotein-cholesterol (LDL-c), with a morbidity of about 1/200 to 1/500. Homozygous familial hypercholesterolemia (HoFH), including compound heterozygous familial hypercholesterolemia and true HoFH, is rare with an incidence of about 1/160,000 to 1/300,000.^[1,2]^ The main pathogenic genes include 3 kinds of genes: low density lipoprotein receptor (LDLR), apolipoprotein B (APOB) and proprotein convertase subtilin/kexin type 9 (PCSK9).^[2]^ In this report, a novel compound heterozygous mutation of LDLR and 2 variants of uncertain significance of APOB and epoxide hydrolase 2 (EPHX2) were detected by pedigree whole exome sequencing (WES), and the related relationship between pathogenic genotypes and clinical phenotypes was further analyzed.

## 2. Case presentation

**Figure 1.**
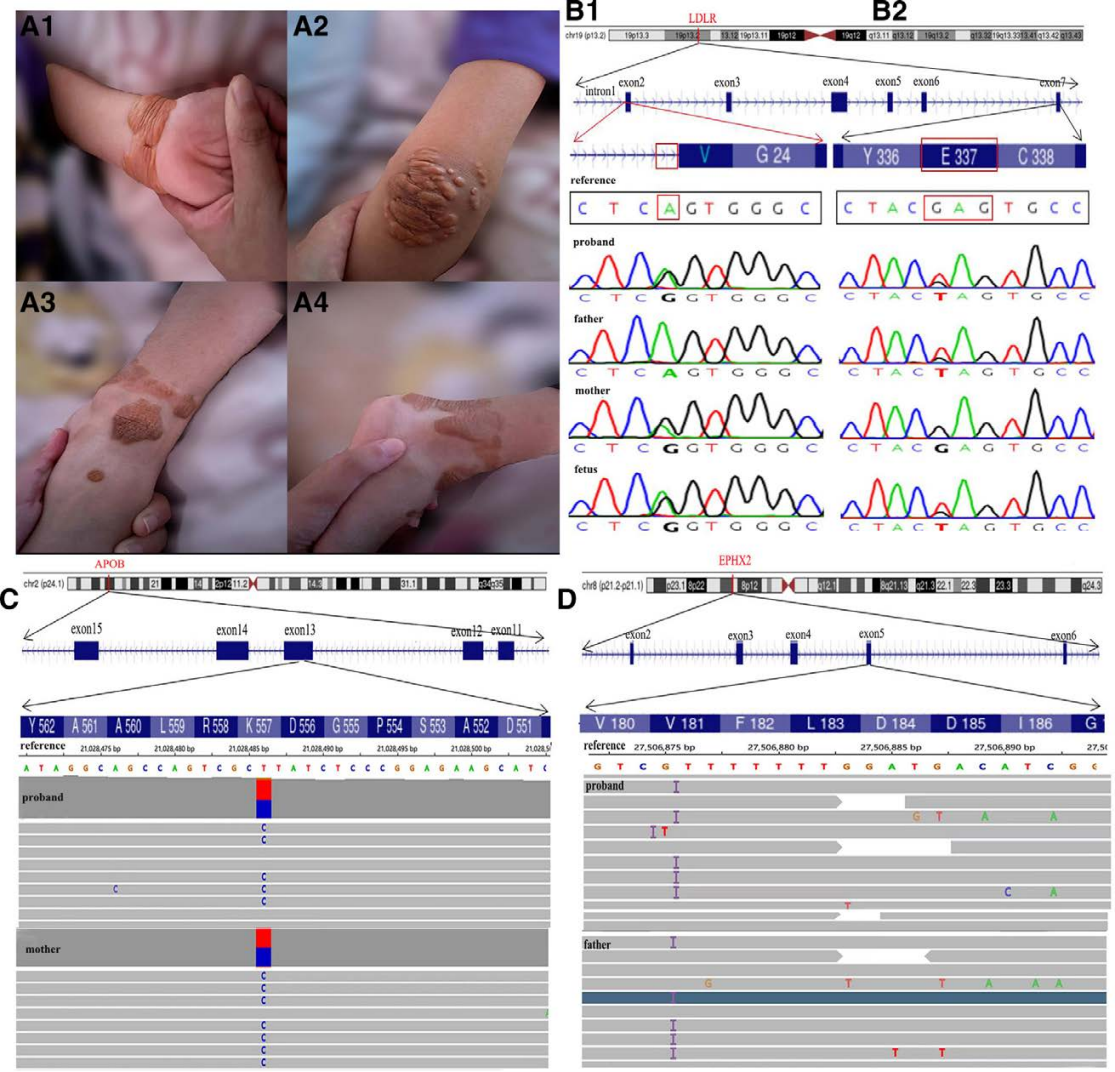
(A) Skin xanthomas: (A1) on the left wrist, (A2) on the left elbow, (A3) and (A4) on the left ankle joint. (B) LDLR gene mutations: (B1) maternal LDLR splice site mutation on chr19: 11100221-11100221: NM_000527.5: c.68-2A>G in intron 1, (B2) paternal LDLR nonsense mutation on chr19: 11110720-11110720: NM_000527.5: c.1009G>T (p.E337X) in exon 7. APOB and EPHX2 of fetus were verified by Sanger sequencing: (C) Maternal APOB missense mutation on chr2: chr2:21028486-21028486, NM_000384.3 complementary DNA sequencing: c.1670T>C in exon 13. (D) Paternal EPHX2 frameshift mutation on chr8:27506875-27506875: NM_001979.6 in exon5. APOB = apolipoprotein B, EPHX2 = epoxide hydrolase 2, LDLR = low density lipoprotein receptor.

**Figure 2.**
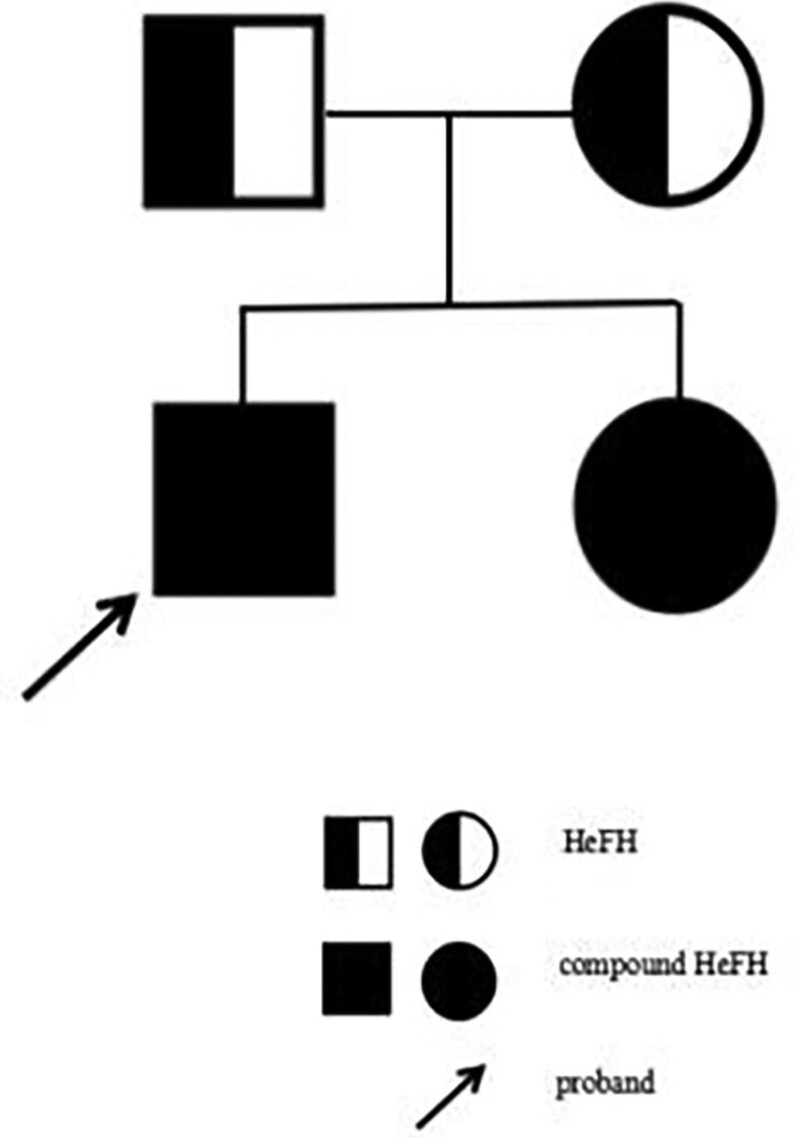
The family tree. HeFH = heterozygous familial hypercholesterolemia.

In June 2018, the proband, a 6-year-old boy, was admitted to the dermatological department of Children’s Hospital Affiliated to the Capital Institute of Pediatrics due to multiple progressive joint skin xanthomas. Blood lipid examination showed significantly elevated cholesterol levels: LDL-c 8.9 mmol/L (normal range [NR]: 2.20–3.60 mmol/L) and total cholesterol (TC) 11.2 mmol/L (NR: 2.80–5.70 mmol/L). APOB variant on chr2: 21028486-21028486, NM_000384.3: c.1670A > G: p.K557R in exon13 (mutation of benign) was detected by trioWES, derived from his mother. He was diagnosed with autosomal dominant familial hypercholesterolemia-2 according to the clinical features and the result of genetic testing. Although diet management and vitamin C tablets were used to control blood lipids and weight, xanthomas on the skin of the whole body were still on the rise trend. Parental blood lipid levels were slightly higher than the upper limit of normal. In August 2022, the mother was referred to Prenatal Diagnosis Medical Center of Xuzhou Central Hospital at 20^+3^ weeks gestation of her second pregnancy. Physical examination of the 10-year-old boy revealed multiple nodular xanthomas of the skin on the wrist, elbow, and ankle (Fig. 1A). The blood lipid levels of 3 family numbers on the same day indicated LDL-c and TC were significantly higher than NR (Table 1). The pedigree WES, including the fetus, was performed again. Source data was obtained via Illumina Novaseq6000 platform according to Berry’s Nano WES Human Exome V1.0 standard operating procedure, and referring to American College of Medical Genetics, Genomics guidelines and Clinical Genome Resource Sequence Variant Interpretation expert groups’ recommendations,^[3,4]^ the pathogenicity of LDLR, APOB, and EPHX2 genes was further evaluated. A compound heterozygous mutation of LDLR gene in the proband was revealed on chr19: 11100221-11100221, c.68-2A>G in intron 1 (NM_000527.5) from mother, and on chr19: 11110720-11110720, c.1009G>T (p.E337X) in exon 7 (NM_000527.5) from father (Fig. 1B1 and B2). In addition, APOB gene on chr2: 21028486-21028486, NM_000384.3: c.1670A>G (p.K557R) in exon 13 (Fig. 1C) and EPHX2 gene on chr8: 27506875-27506875, NM_001979.6: c.548dup (p.L183Ffs*3) in exon5 (Fig. 1D) of the proband were found from the mother and the father, respectively. The same compound heterozygous mutation of LDLR gene was verified in the fetus (Fig. 1B1 and B2), while no abnormalities of APOB and EPHX2 variants by sanger sequencing, and no chromosomal or structural anomalies by karyotyping, copy number variation sequencing and systemic ultrasound were found. After genetic counseling, the parents decided to continue the pregnancy. On the following December 26, 2022, a baby girl was born, weighing 4.05 kg without skin xanthomas, and her blood lipid level at birth was within the NR (Table 1). According to physical, biochemical examinations and genetic tests, the pedigree was diagnosed with FH. The family tree was as shown in Figure 2. After that, treatment advice and follow-up were offered to them: a healthy diet (less high-fat and high-cholesterol foods), proper physical exercise and weight control, and a consultation with cardiovascular specialist were recommended. During the follow-up in May 2023, we were told that the brother’s skin xanthomas were well controlled, although the blood lipids were still higher than NR, while the younger sister was well-fed and of normal weight: 8 kg at 5 months old. However, cardiovascular function examinations for the family were not performed regularly. This study was approved by Xuzhou Central Hospital Ethics Committee. Informed consent was obtained from the parents for the publication of our case.

**Table 1:** Blood lipid levels of the family members.

Blood lipid levels	Brother	Father	Mother	Sister
LDL-c (NR: 2.20–3.60 mmol/L)	18.00	6.87	5.42	2.80
TC (NR: 2.80–5.70 mmol/L)	19.40	10.18	8.63	4.50
HDL-c (NR: 0.80–1.70 mmol/L)	1.10	0.91	2.36	0.90
Triglyceride (NR: 0.45–0.75 mmol/L)	1.08	6.04	1.26	0.65

HDL-c = high density lipoprotein-cholesterol, LDL-c = low density lipoprotein-cholesterol, NR = normal range, TC = total cholesterol.

## 3. Discussion

The compound heterozygous LDLR mutation is relatively rare in the patients with FH. In our report, 2 babies in 1 family carried the same novel compound heterozygous LDLR mutation. According to the American College of Medical Genetics, Genomics and ClinGen guidelines,^[3,4]^ compound heterozygous LDLR mutations: exon7: c.1009G>T (p.E337X) and intron1: c.68-2A>G both own evidence of strong pathogenicity (PVS1), were confirmed as functional loss variants resulting in functional changes of the LDLR protein. Both of them, classified as class I mutations (nonsense and splice site mutations), have been confirmed to prevent LDLR protein from being synthesized due to an advanced stop codon and to prevent intravascular LDL from entering the cell and being cleared by lysosome. Moreover, the frequencies of the mutations were below 0.0002 in Genome Aggregation Database (gnomAD) or other large populations (PM2) and constituted compound heterozygote (PM3). Hence, the compound heterozygous LDLR has been certified as a pathogenic mutation and the patients usually present higher blood LDL levels.^[2]^ In addition, the APOB variant in exon13: c.1670A>G (p.K557R), a missense mutation, might aggravate defective expression of apolipoprotein B-100 (apoB100), impair LDL-LDLR binding, and affect the clearance of LDL-c in the vessel.^[5]^ Therefore, the proband, his parents and sister were diagnosed with FH combined with the main phenotypes, genetic tests (Fig. 1) and the results of lipid analysis (Table 1), according to the Dutch Clinical Lipid Network (LDL-c level ≥ 4.0 mmol/L, score ≥ 1; the pathogenic mutation of any gene LDLR, APOB or PCSK9 manifested by 8 points: score > 8 can be diagnosed as FH) and the 2015 American Heart Association expert consensus (genetic testing including pathogenic mutations as the gold standard for the diagnosis of FH).^[2,6]^

Cholesterol is involved in a variety of important physiological processes. In FH, multi-gene mutations such as LDLR and APOB could lead to dysregulation of cholesterol metabolism and result in ASCVD. The histiocytes and macrophages that engulf LDL-c accumulate in the subcutaneous area, forming skin xanthoma or tendon xanthoma.^[5]^ By comparing the results of blood lipids of family members, the LDL-c level of the mother was lower than that of other members, and high density lipoprotein-cholesterol level was higher than NR, which might be related to the increase of estrogen during pregnancy.^[7]^ In addition, the EPHX2 variant of the proband and his father: exon 5: c.548dup: P.183FFS*3, a frame-shifted mutation, was revealed. Sato et al once reported a missense mutation of EPHX2 gene in 2004: p.Arg287Gln, which could modify the LDLR variants and affect the phenotypes, especially serum triglyceride levels.^[8]^ Both father and mother had pathogenic mutations of LDLR, but the triglyceride level of father is significantly higher than that of mother, which may be associated with EPHX2 variant. Moreover, the results of 2 genetic tests were different, which might be related to the progress of molecular genetic technology and the depth of sequencing. Therefore, the same test method applied to the same patient in different periods may have different results, and should be evaluated from a dynamic and critical point of view, especially in the case of inconsistent genotype and phenotype, it should be treated with caution.

A combination of diet and medication has been recommended for the patients with FH, and cardiovascular risk assessment should be accompanied by physical exercise. Individualized treatment education for patients with FH could help improve compliance, reduce anxiety, and improve long-term quality of life.^[2,6]^ The ideal LDL target for FH in childhood is below 3.5 mmol/L, or 30% to 50% lower than the base line value.^[9]^ However, for children below 1 year old, dietary fat is important for brain and cognitive development in infancy and should not be restricted.^[10]^ Furthermore, the proband was in a critical period of growth and development. Under the premise of ensuring nutritional need for growth and development, it is necessary to limit the intake of cholesterol and saturated fatty acids, conduct appropriate physical exercise, control weight, measure blood lipid levels, and take statins when appropriate.^[9]^ Statin therapy should be considered when the patient is 8 years old or older while treatment needs to be initiated at an earlier age if ASCVD is accompanied or HoFH is diagnosed.^[9]^ Relevant studies have proved that statins have no negative effects on the growth and development of children in the short term, but the long-term effects are still unclear.^[10]^ Several countries have recommended that statins be started at low doses.^[9]^ In addition, patients with LDL-c > 10 mmol/L can undergo lipoprotein apheresis as early as possible (5–8 years old).^[2]^ Treatment and management for the parents of the proband: the ideal serum LDL target should be <2.6 mmol/L, and a healthy lifestyle such as quitting smoking and alcohol cessation should be required. After cardiovascular risk is eliminated, active physical exercise and weight control are recommended. Eat more vegetables, fruits, fish, whole grains and less high-fat, high-cholesterol foods, and use statins, ezetimibe or PCSK9 inhibitors in combination.^[6]^

## 4. Conclusions

In summary, genetic analysis combined with clinical features is essential for the diagnosis of FH. Furthermore, 2 children with compound heterozygous familial hypercholesterolemia in 1 family may have a certain impact on them during their developmental period. Therefore, it was significant to provide the guidance and early intervention for such families in the long run.

## Acknowledgments

We would like to thank for the families’ participation and the staffs’ cooperation of the department of Prenatal Diagnosis Medical Center of Xuzhou Central Hospital.

## Author contributions

**Conceptualization:** Yu Han, Lin Zhang, Jingfang Zhai.

**Data curation:** Lin Zhang, Jingfang Zhai.

**Investigation:** Huimin Tao, Jiebin Wu.

**Supervision:** Jingfang Zhai.

**Writing – original draft:** Yu Han.

**Writing – review & editing:** Huimin Tao, Jiebin Wu, Jingfang Zhai.
